# Preterm Birth and the Emergence of ADHD Symptoms: A Review of Recent Evidence

**DOI:** 10.3390/medicina62010024

**Published:** 2025-12-23

**Authors:** Panagiotis Papanikolopoulos, Stavroula Papanikolopoulou, Angeliki Gerede

**Affiliations:** 1 Latsia Health Center, CY-1452 Nicosia, Cyprus; 2 First Department of Obstetrics and Gynecology, Medical School, Aristotle University of Thessaloniki, GR-54124 Thessaloniki, Greece; papanikolopoulous@gmail.com; 3 Unit of Maternal-Fetal-Medicine, Department of Obstetrics and Gynecology, Medical School, Democritus University of Thrace, GR-68100 Alexandroupolis, Greece; agerede@otenet.gr

**Keywords:** ADHD symptoms, neurodevelopmental disorder, preterm birth, prematurity

## Abstract

*Background and Objectives*: Preterm birth is a common obstetric problem. Attention-deficit hyperactivity disorder (ADHD) affects an increasing number of children. There is evidence that all subcategories of preterm birth are related to the occurrence of ADHD. The present article reviews the findings of the last two years regarding this association. *Materials and Methods*: PubMed was screened for relevant articles published in English between January 2024 and November 2025. Keyword combinations of the words “preterm birth”, “prematurity”, “attention deficit disorder”, “attention disorders”, “ADHD”, “preterm” and “attention deficit hyperactivity disorder” were used. A total of 28 articles were retrieved, reviewed and selected. *Results*: Preterm birth results in higher risk of ADHD, while early preterm births are characterized by an even higher risk of ADHD. Additionally, postnatal complications commonly experienced by preterm infants are associated with the presence of ADHD. It seems that the maternal use of ADHD medications during pregnancy is associated with a high risk of preterm birth, although there is a concern about the role of other psychotropic medications during pregnancy. Various neurodevelopmental disorders are also associated with preterm birth. Maternal use of glucocorticoids combined with preterm birth leads to higher risk of ADHD. However, the outcome of ADHD is shaped by a wide range of social, familiar and biological factors. *Conclusions*: Prematurity is a significant risk factor for the development of ADHD symptoms in children. However, many biological, environmental, and psychosocial factors, such as neurodevelopmental vulnerability, perinatal complications, maternal health and adverse psychosocial factors, act as regulators in this relationship. Researching and understanding these associations will help in implementing preventive measures in children who are at increased risk of developing ADHD.

## 1. Introduction

Preterm birth remains a worldwide health concern because of ongoing challenges in prediction and prevention. Current predictors are characterized by poor performance, need for invasive sampling, and an inability to identify patients in a timely fashion to allow for effective intervention [1].

Attention-deficit hyperactivity disorder (ADHD) is a common childhood neurodevelopmental and behavioral disorder. At least 5% of children have substantial difficulties with overactivity, inattention, and impulsiveness that are just under the threshold to meet full diagnostic criteria for ADHD. The prevalence (clinically diagnosed or recorded) of ADHD varies worldwide and has been increasing over time [2].

Gestational age is defined as the length of pregnancy from the beginning of the mother’s last menstrual period to the birth delivery date. Gestational age has been further categorized by the World Health Organization as extremely preterm (<28 weeks), very preterm (28 to less than 32 weeks), moderate to late preterm (32 to 37 weeks), term (37–41 weeks), and postterm (>41 weeks). Children born preterm in all subcategories, from extremely preterm to late preterm, have an increased risk of developing ADHD [3]. However, it is unclear to what extent this association can be explained by shared genetic and environmental risk factors and whether gestational age at birth is similarly related to inattention and hyperactivity/impulsivity [4]. These findings suggest that preventive interventions in clinical care and educational settings may be essential in improving outcomes for preterm-born children at risk for ADHD.

The objective of this review is to examine the association between preterm birth and the occurrence of ADHD, and it synthesizes recent findings (published papers from 2024 to 2025) about the association between preterm birth and the development of ADHD symptoms in childhood and adolescence. Drawing results from large-scale cohort studies, meta-analyses, and neurodevelopmental investigations, it explores biological, environmental, and perinatal mechanisms that may underline this link.

## 2. Materials and Methods

This narrative review was conducted based on a structured literature search across PubMed. The goal was to identify peer-reviewed studies examining the relationship between preterm birth and attention-deficit/hyperactivity disorder (ADHD), including potential influencing factors. The keyword combinations used were (“preterm birth” OR prematurity) AND (“attention deficit disorder” OR “attention disorders” OR ADHD), (“preterm birth” AND ADHD) OR (prematurity AND ADHD) and (“preterm birth” OR preterm) AND “attention deficit hyperactivity disorder”.

This research focused on articles written in English and published during the last two years (2024–2025) to capture the most current evidence on the topic. Included articles were original research, systematic or narrative reviews, and meta-analyses that directly or indirectly examine the relationship between prematurity and ADHD, and/or report influencing factors. Excluded were case reports and conference abstracts.

The selection process followed PRISMA guidelines. After deduplication, titles and abstracts were screened for relevance. Full-text eligibility was then assessed by two independent reviewers. Discrepancies were resolved by discussion. A PRISMA-like flowchart illustrating the article selection process is provided in Figure S1.

The findings were synthesized narrative to highlight current knowledge, identify research gaps, and inform future clinical practices.

## 3. Epidemiological Evidence Linking Preterm Birth and ADHD

A Swedish national registry study by Crump et al. found that preterm birth is associated with a modestly increased risk of ADHD [5], while early preterm births are characterized by a significantly higher risk [6].

Epidemiological studies have consistently demonstrated a strong association between preterm birth and increased risk of ADHD. Balit et al. present evidence from a large registry-based linkage cohort study showing that individuals born preterm have significantly increased rates of psychiatric disorders, including ADHD, compared with those born at term [7]. These findings suggest that preterm birth is a perinatal risk factor with adverse neurodevelopmental consequences. Also, Kong et al. demonstrate a strong association between preterm birth and later ADHD and psychiatric disorders in a large birth cohort [8].

Importantly, the risk remained after controlling for siblings (controlling for common familial confounders), strengthening the hypothesis of a causal relationship between gestational age and ADHD [6]. This interaction highlights the important role of social factors as a predictor of the occurrence of ADHD, suggesting that both perinatal and environmental factors contribute to the risk of ADHD.

A meta-analysis by Zhao et al. supports these associations by synthesizing findings from multiple studies [9]. The analysis reveals that preterm birth is a significant predictor of ADHD, particularly when combined with low maternal education.

Guedria et al. identified premature birth as a significant risk factor for ADHD in a Tunisian child population, noticing the contribution of the economic status (low- and middle-income) [10].

Similarly, Zumbach-Basu et al. provide evidence linking prematurity and fetal growth restriction to increased levels of hyperactivity, inattention, and externalizing behaviors such as aggression and delinquency [11]. These effects persist across developmental stages, highlighting the long-term behavioral and psychiatric consequences of preterm birth.

## 4. Perinatal Factors Influencing the Risk of ADHD

Perinatal factors, particularly those associated with preterm birth, such as a history of preterm delivery, hypertensive disorders, inadequate antenatal care compliance, and postnatal complications, play a critical role in shaping neurodevelopmental difficulties, including the risk for attention deficit/hyperactivity disorder. An extensive body of research highlights how both biological and pharmacological exposures during the perinatal period contribute to these risks.

Srinivas et al. link maternal use of ADHD medications during pregnancy to an increased risk of preterm birth [12]. While these medications may be clinically necessary, their association with preterm pregnancy is of concern, as such pregnancies are an established risk factor for ADHD. This finding highlights the importance of decisions made during pregnancy, as the mental health needs of the mother must be carefully balanced with the potential developmental effects on the child.

The topic seems to be complex, as Fabiano et al. refer to mixed results regarding the safety of psychotropic medications during pregnancy [11]. In some cases, there is an increased risk of adverse perinatal complications, including preterm birth, across several drug classes. Therefore, it is crucial to assess drug exposure as a modifiable perinatal risk factor that may indirectly influence the susceptibility of the newborn to ADHD.

In addition to pharmacological factors, Tso et al. examined how postnatal complications commonly experienced by preterm infants—such as respiratory distress, intra-abdominal hemorrhage, and sepsis—are significantly associated with an increased risk of ADHD [3]. Their findings suggest that the neurodevelopmental consequences of preterm birth are not solely attributable to gestational age but also to the medical complications during the neonatal period. This evidence supports a developmental framework in which prematurity and perinatal complications interact to shape long-term behavioral developmental disorders.

## 5. Prematurity and Neurodevelopmental Disorders

Angel et al. found a modestly increased risk of ADHD and other neurodevelopmental disorders among assisted reproductive technologies (ARTs)—conceived children, largely mediated by higher rates of preterm birth and low birth weight in this group [13].

Chang et al. conducted a retrospective national cohort study in Taiwan, which found that in vitro fertilization IVF strategies that lead to multiple pregnancies and often result in preterm birth were associated with an increased risk of neurodevelopmental disorders, like ADHD [14].

Extremely preterm children with severe retinopathy of prematurity were also at elevated risk of neurodevelopmental autism and other neurodevelopmental disorders [15].

According to the study by Nivins et al., neural dysfunctions differ by degree of prematurity [16]. It was found that impairments in preschool executive functions are partially associated with preterm birth. These findings suggest that executive functioning deficits serve as a developmental mechanism linking preterm birth to later attentional difficulties.

Various attention problems among preschool-aged children born very preterm were identified by a study of Camerota et al. [17]. Neonatal complications and socio-demographic risk factors were associated with more severe attention problems.

At the same time, the study of Moriichi et al. reveals that the coexistence of ADHD in adolescents with a history of extremely low birth weight is associated with disorders of mental health and social adaptation [18]. These findings reinforce the view that ADHD, when manifested in the context of premature birth, is not just a neurodevelopmental disorder, but a psychosocial factor that worsens the already precarious psychosocial course of these children.

Finally, history of meningitis [19] or history of periviable rupture of membranes [20] with preterm birth are reported as factors that may increase the risk of neurodevelopment problems.

## 6. Inflammatory and Hormonal Mechanisms

Friedman et al. suggest that chronic inflammation and the maternal immune activation potentially influence fetal brain development and are related to preterm birth, which in turn increases the risk for neurodevelopmental consequences, including ADHD [21].

Prenatal exposure to systemic glucocorticoids increases the risk of ADHD (and other mental and neurodevelopmental problems) of exposed offspring [18,22], but according to Ho et al. this high risk is observed exclusively in preterm births, while full-term pregnancies are not affected [22].

By the same mechanism maternal perinatal depression is related to stress-related hormonal changes (e.g., elevated cortisol) contributing to preterm and ADHD pathogenesis [23].

Finally, there is evidence of an association between gestational diabetes mellitus(GDM) and ADHD, but further research is needed [24].

## 7. Socioenvironmental and Genetic Modulators

Preterm birth is associated with an increased risk of ADHD symptoms during childhood and adolescence, but the outcome of the disorder is shaped by a wide range of social, familiar and biological factors. According to Gurgel et al., psychiatric disorders of parents, family history of ADHD and socio-economic conditions are important predictors of early ADHD diagnosis in preschool age [25]. These factors enhance the vulnerability of children who already have a biological risk due to premature birth, underlining the importance of the social context.

It is noteworthy that, according to the study of Tusa et al., perinatal maternal depression elevates the risk of offspring ADHD, independently of adverse birth outcomes, indicating maternal depression as an important predisposing factor for the occurrence of ADHD, independently of the perinatal complications [23].

Maternal ADHD is related to higher possibility of perinatal complications, especially preterm birth and low birth weight [26]. However, according to Andersson et al. there is no correlation between maternal ADHD and preterm birth [27], while Song et al. report modestly increased rates of preterm birth among pregnant women with ADHD [28].

Children born very prematurely were exposed to more Adverse Childhood Experiences and had more severe ADHD and internalizing problems than full-term children. This difference remained even after controlling for confounding factors [29].

A large meta-analysis at the pan-European level showed that deviations in early development are associated with an increased risk of mental health problems, including ADHD features as well as intellectual potential. It is particularly important that socio-economic and environmental factors, such as the family environment and the level of care, act as regulators of the relationship between early growth and neurodevelopment outcomes in children [30].

Changes in the number of copies of one or more parts of the genome are associated with a range of developmental deficits, including ADHD. They are also associated with a greater likelihood of prematurity, motor delay, coordination and speech problems, and more generally special educational needs, i.e., problems that often coexist with ADHD [31].

The study by Gurgel et al. showed many signs that can help find ADHD in young kids [25]. Lots of these signs are linked to family problems and biological issues. For example, early contractions during pregnancy and stomach reflux were found to raise the risk showing how important it is to look at struggles during pregnancy. Special focus was put on parents’ signs like past mental health issues or being in trouble with the law, which may show some genetic load or troubles at home. Alongside this, having low money status for the family was tied to a higher chance of getting diagnosed and having lasting symptoms of ADHD. These results back up the idea that both genes and social factors work together to affect how children might have biological issues, such as those born prematurely.

## 8. Discussion

It seems that preterm birth is associated with an increased likelihood of developing ADHD. This clinical finding underscores the need for early recognition, regular follow-up, and timely intervention, particularly in those cases with additional perinatal, social, or environmental problems. The use of glucocorticoids or ADHD medication during pregnancy should be applicable very carefully, as these conditions are associated with high risk of ADHD. Therefore, their coexistence with preterm birth will lead to serious risk of occurrence of ADHD. The need for increased developmental surveillance in preterm infants may facilitate early diagnosis and intervention, potentially mitigating long-term behavioral and developmental (cognitive, speech, or motor) consequences.

Women’s mental health care during and after pregnancy is very important, given the well-documented associations between maternal depression and stressors (metabolic and environmental) with the risk of ADHD. This requires collaboration between obstetricians, pediatricians, child psychiatrists, and social workers to develop comprehensive care protocols that address both biological and psychosocial risk factors.

Research findings suggest the potential for targeted interventions in high-risk populations. These may include parent education programs, early behavioral therapies, and psychoeducation regarding children’s neurodevelopmental outcomes. Future research should continue to explore the mechanisms underlying the association of early pregnancy with the onset of ADHD through longitudinal studies that include adolescence and adulthood. Investigation of protective factors and mechanisms of resilience may also provide insights into mitigating the severity of ADHD and other neurodevelopmental disorders in this vulnerable population.

There are a number of limitations to this review. First, the literature search was limited to English-language studies available in the PubMed database, potentially omitting studies that were indexed in other databases or published in other languages. Second, the articles reviewed reflected a high level of heterogeneity in study designs, definitions of prematurity, age at assessment and diagnostic criteria for ADHD, making direct comparisons between study data not easily feasible. Third, many of the studies used administrative health records that could underrepresent or overrepresent subclinical or undiagnosed cases. In addition, not all studies accounted well for potential confounders (e.g., socioeconomic status, perinatal complications or parental mental health) that might also impact the relationships strongly. Finally, studies on assisted reproductive technologies, prenatal exposures, and neonatal complications rarely addressed specific biological mechanisms and rarely included comprehensive long-term developmental follow-up.

Despite these limitations, our analysis revealed a number of important knowledge gaps. Firstly, the majority of the studies related to this topic have been conducted in well-developed countries. So, there is a gap in this correlation in lower-income countries. Second, IVF strategies are correlated with an increased risk of neurodevelopmental disorders, but there is a gap in whether this relationship is related to the increased risk of preterm birth that accompanies multiple pregnancies or whether there is an independent association. Third, most studies do not investigate the role of the severity of prematurity in the occurrence of ADHD. It is important to know the differences in the likelihood of developing ADHD between extremely preterm, very preterm and moderate to late preterm birth.

## 9. Conclusions

This review, based on various studies from the recent literature, highlights prematurity as a significant risk factor for the development of ADHD symptoms in children. This relationship is also influenced by a multitude of other biological, environmental, and psychosocial factors, such as neurodevelopmental vulnerability, perinatal complications, maternal health and adverse psychosocial factors.

Recognizing prematurity as a key predictor of offspring with ADHD underscores the need for effective interventions. Ongoing research into modifiable risk factors and protective factors, as well as the developmental trajectory of these children, is crucial. It is important to conduct studies that will document differences in the risk of ADHD between degrees of prematurity. Understanding the importance of the above factors, as well as early and multifaceted interventions, can contribute to improving mental health of children and their families, both in the short and long term.

## Data Availability

No new data were created or analyzed in this study. Data sharing is not applicable to this article.

## Supplementary Materials

The following supporting information can be downloaded at: https://www.mdpi.com/article/10.3390/medicina62010024/s1, Figure S1: Flow diagram illustrating the study selection process [32].

## Abbreviations

The following abbreviations are used in this manuscript:

ADHD	Attention-deficit hyperactivity disorder
ARTs	Assisted reproductive technologies
GDM	Gestational diabetes mellitus
