# Supplementary material for: Preterm Birth and the Emergence of ADHD Symptoms: A Review of Recent Evidence
Source: Medicina (Kaunas). 2025 Dec 23;62(1):24. doi: 10.3390/medicina62010024 (PMC12842838; doi:10.3390/medicina62010024)
Supplement: Supplementary file 1 [file medicina-62-00024-s001.zip › medicina-4038399-supplementary.docx]

**Identification of studies via databases**

Following database searches, records were deduplicated

(n=68)

Records identified from

Pubmed (n =154)

**Identification**

Reports excluded due to not meeting inclusion criteria or lacking relevance to prematurity and ADHD.

(n=58)

Reports assessed for eligibility

(n =86)

**Screening**

Studies included in review

(n = 28)

**Included**

*Consider, if feasible to do so, reporting the number of records identified from each database or register searched (rather than the total number across all databases).

**If automation tools were used, indicate how many records were excluded by a human and how many were excluded by automation tools.

*From:*  Page MJ, McKenzie JE, Bossuyt PM, Boutron I, Hoffmann TC, Mulrow CD, et al. The PRISMA 2020 statement: an updated guideline for reporting systematic reviews. BMJ 2021;372:n71. doi: 10.1136/bmj.n71

For more information, visit:<http://www.prisma-statement.org/>
